# Rapid glutamate receptor 2 trafficking during retinal degeneration

**DOI:** 10.1186/1750-1326-7-7

**Published:** 2012-02-10

**Authors:** Yanhua Lin, Bryan W Jones, Aihua Liu, Félix R Vazquéz-Chona, J Scott Lauritzen, W Drew Ferrell, Robert E Marc

**Affiliations:** 1 Department of Ophthalmology, John A. Moran Eye Center, University of Utah School of Medicine, 65 Mario Capecchi Drive, Salt Lake City, UT 84132, USA

**Keywords:** glutamate receptor 2, retinal degeneration, retinal remodeling, neuritogenesis

## Abstract

**Background:**

Retinal degenerations, such as age-related macular degeneration (AMD) and retinitis pigmentosa (RP), are characterized by photoreceptor loss and anomalous remodeling of the surviving retina that corrupts visual processing and poses a barrier to late-stage therapeutic interventions in particular. However, the molecular events associated with retinal remodeling remain largely unknown. Given our prior evidence of ionotropic glutamate receptor (iGluR) reprogramming in retinal degenerations, we hypothesized that the edited glutamate receptor 2 (GluR2) subunit and its trafficking may be modulated in retinal degenerations.

**Results:**

Adult albino Balb/C mice were exposed to intense light for 24 h to induce light-induced retinal degeneration (LIRD). We found that prior to the onset of photoreceptor loss, protein levels of GluR2 and related trafficking proteins, including glutamate receptor-interacting protein 1 (GRIP1) and postsynaptic density protein 95 (PSD-95), were rapidly increased. LIRD triggered neuritogenesis in photoreceptor survival regions, where GluR2 and its trafficking proteins were expressed in the anomalous dendrites. Immunoprecipitation analysis showed interaction between KIF3A and GRIP1 as well as PSD-95, suggesting that KIF3A may mediate transport of GluR2 and its trafficking proteins to the novel dendrites. However, in areas of photoreceptor loss, GluR2 along with its trafficking proteins nearly vanished in retracted retinal neurites.

**Conclusions:**

All together, LIRD rapidly triggers GluR2 plasticity, which is a potential mechanism behind functionally phenotypic revisions of retinal neurons and neuritogenesis during retinal degenerations.

## Background

Retinal degenerations (RD), such as age-related macular degeneration (AMD) and retinitis pigmentosa (RP), are progressive disorders initiated by photoreceptor stress and are accelerated by photoreceptor death, which effectively deafferents the inner retina and evolves into formal retinal remodeling [1-3]. Thus, retinal remodeling proceeds through three phases: 1, photoreceptor stress; 2, photoreceptor death and 3, complex neural remodeling [3]. Two of the major hallmarks of retinal remodeling are growth of novel neurites and functional reprogramming of existing retinal neurons [1-8]. Pathogenic neuronal reprogramming and de novo neuritogenesis are not isolated to retinal tissues, as pathological revision also occurs in neurodegenerative diseases such as epilepsy [9] and Alzheimer's disease [10]. Retinal remodeling limits the effectiveness of vision rescue strategies including photoreceptor- and retinal pigment epithelium (RPE)-directed therapies [4,6,7,11,12]. Better understanding of the mechanisms underlying retinal remodeling will improve the outcomes of genetic, molecular, cellular and bionic rescues.

Deafferentation of the neural retina eliminates the intrinsic glutamatergic drive by the sensory retina [3] and induces glutamate receptor reprogramming before gross topologic restructuring of the retina begins [4,13]. In phase 2 degenerating retinas with extensive rod death, the downstream rod-specific signaling pathways persists [13,14], and bipolar cells still respond to glutamate receptor agonists [4,7,15]. Among the glutamate receptors (GluRs), α-amino-3-hydroxy-5-methyl-4-isoxazolepropionic acid (AMPA) receptors mediate fast synaptic transmission at excitatory synapses in CNS and are tetrameric assemblies of subunits GluR1-4 encoded by separate genes [16]. Their involvement and modulation during neuronal development, synaptic plasticity and structural remodeling is fundamental to timing and coherence of developing neural networks [17]. In brain, combined neuronal activity and pathologic insults trigger rapid changes in postsynaptic AMPA receptor attributes (e.g. subunit composition) and may control Ca^2+ ^permeability [18]. Ca^2+ ^fluxes play critical roles in neural function, including the regulation of neurite outgrowth and synaptogenesis [19], synaptic transmission and plasticity [20], and cell survival [21]. GluR2 in heteromeric AMPARs renders the channel low permeable to Ca^2+ ^[22,23], so that even a modest alteration in the level of GluR2 is expected to have profound implications for synaptic efficacy and neuronal survival [24].

Given prior evidence of iGluR reprogramming in human RP and animal models of RD [4,8,25], we hypothesized that retinal iGluRs, especially GluR2 subunits are modulated in retinal degenerative diseases. GluR2 subunit expression is associated with vertical channel retinal processing [26-28], and its expression limits AMPAR permeability to Ca^2+ ^[29]. In this sense it is thought to be neuroprotective [30,31]. To study the kinetics of GluR2 expression and trafficking in retinal degenerative disease, we used the LIRD model, which contains the full spectrum of sequelae found in naturally occurring and engineered forms of retinal degeneration and remodeling, including early retinal stress, photoreceptor loss, Müller cell remodeling, neuritogenesis [8], and remodeling of all neural cell populations in the retina and formation of microneuromas [8,12]. Our analysis of glutamate receptors and neuritogenesis in the light-damage model spans phases 1 and 2. This work demonstrated that in a LIRD model, GluR2 levels and trafficking rapidly increased in response to light-induced photoreceptor stress and death, providing a potential feedback mechanism for controlling Ca^2+ ^permeability in retinal neurons. Most importantly, GluR2 upregulation may occur in ON bipolar cells, which are normally hyperpolarized by glutamate. Expression of AMPA receptors would change their polarity as predicted by Marc et al 2007 [4] and Jones et al. [13] in mouse, rabbit and human retina. In addition, the motor protein KIF3A colocalized well with PSD-95 and GRIP1 at novel sprouting neurites, potentially indicating a chaperone role for KIF3A, guiding GluR2 and its trafficking proteins to newly forming dendritic processes.

## Results

### LIRD increases GluR2 expression prior to obvious photoreceptor loss

**Figure 1:**
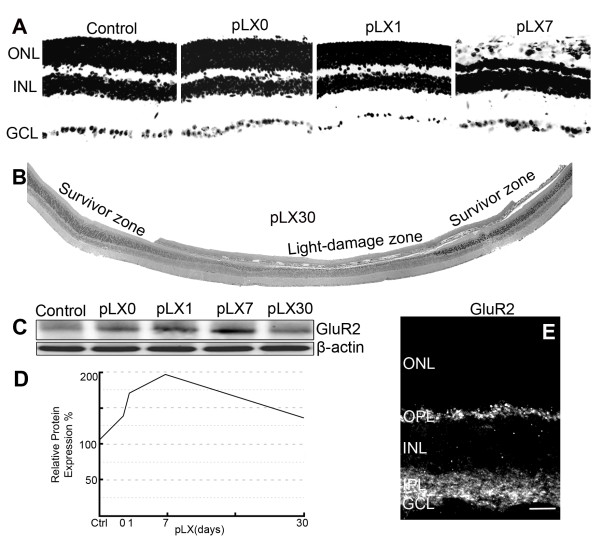
**Light stress induced photoreceptor degeneration and increased GluR2 levels**. **A**, **B**. DAPI staining showed that light stress dramatically led to photoreceptor loss by pLX7. The whole retina thus was differentiated into light-damage and survivor zones. Western blotting (**C**) and analysis (**D**) showed increased levels of GluR2, n = 9. **E**. Confocal imaging revealed expected GluR2 expression in the OPL and IPL of control retina (postnatal day 80); scale = 20 μm.

LIRD led to dramatic photoreceptors loss by post-light exposure day 7 (pLX7) (Figure 1A), demonstrating that LIRD is a "fast degenerating" animal model of retinal degeneration [32]. Consistent with our previous results [8,12], light stress differentiated the mouse retina into survivor zones, where stressed photoreceptors and retinal neurons persisted, and light-damage zones, where rods and cones died (Figure 1B). Despite that DAPI staining showed a normal thickness of inner nuclear layer (INL) in both the survivor and light-damage zones and histological analysis of retina revealed normal lamination and topology at light onset (pLX0) through pLX1 (Figure 1A and 1B), protein levels of GluR2 showed immediate response to light stress. Specifically, protein levels of GluR2 increased immediately at pLX0, peaked at pLX7, and recovered to almost control levels by pLX30 (Figure 1C and 1D). GluR2 immunostaining of control tissues (postnatal day 80) revealed GluR2-immunoreactive puncta aggregating in the outer plexiform layer (OPL) with more GluR2-immunoreactive puncta found throughout the OFF and ON sublamina (Figure 1E).

### GluR2 trafficking protein levels are modulated during LIRD

**Figure 2:**
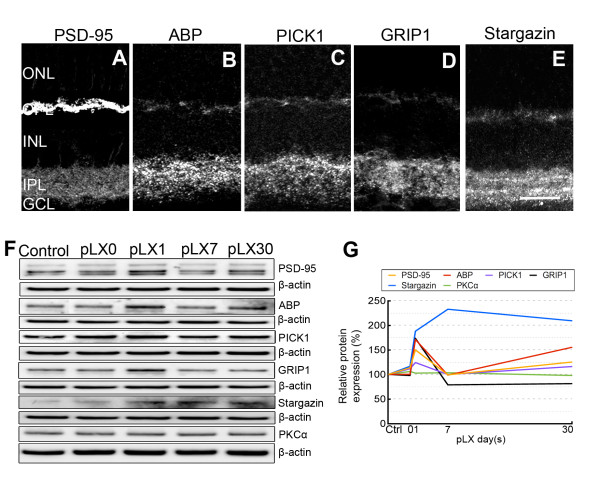
**GluR2 trafficking proteins were modulated during LIRD**. Confocal imaging showed that PSD-95 (**A**), ABP (**B**), PICK1 (**C**), GRIP1 (**D**), and stargazin (**E**) were expressed in the OPL and IPL (postnatal day 80). Western blotting (**F**) and analysis (**G**) showed that protein levels of PSD-95, ABP, GRIP1, and stargazin were increased, while levels of PICK1 and PKCα showed no change, n = 9; scale = 20 μm.

GluR2 is assembled, with other GluR subunits, into iGluR channels in the postsynaptic membrane [33]. The C-terminus of GluR2 contains a PDZ domain that binds GluR2 to trafficking proteins including GRIP1, ABP, PICK1 [34-36], and stargazin [37]. GluR2 is anchored at synaptic and intracellular membranes by ABP/GRIP but cycles between these membranes in association with PICK1 [38]. Consistent with a previous report [39], PSD-95 was most prominent in the rod spherules and cone pedicles of the OPL, weak PSD-95 labeling was also present in the inner plexiform layer (IPL) (Figure 2A). The retinal distributions of GluR2 trafficking proteins GRIP1, ABP, PICK1 and stargazin showed labeling in the OPL and IPL (Figure 2B-E). These data confirm the presence of GluR2-related PSD proteins such as ABP and GRIP1 in the retina as demonstrated by Gabriel et al [40]. Similar to findings in GluR2 protein expression, PSD-95, GRIP1, ABP, and stargazin expression were increased by light stress, levels of PSD-95, ABP, and GRIP1 peaked at pLX1 and then recovered, while the levels of stargazin were elevated from pLX1 to pLX30, the last time point in the experiment (Figure 2F and 2G). Protein levels of PKCα and PICK1 were unaltered by light exposure from pLX1 to pLX30 (Figure 2F and 2G). Western blotting images of PSD-95 protein levels revealed three adjacent bands (Figure 2F), potentially due to that the anti-PSD-95 IgG targets a common epitope in the homologous regions [39].

### LIRD triggers neuritogenesis by rod bipolar cells and horizontal cells in the survivor zone

**Figure 3:**
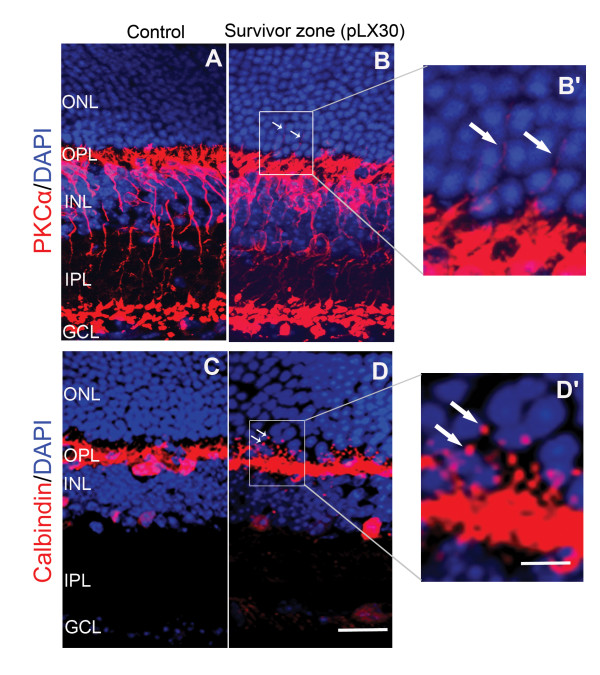
**LIRD induced retinal neuritogenesis**. **A**, **B**. PKCα immunohistochemistry revealed that LIRD induced neuritogenesis by rod bipolar cells, with dendrites extending from the OPL into the ONL (highlighted by arrows). **C**, **D**. Calbindin immunohistochemistry showed neuritogenesis by horizontal cells with processes extending from the OPL into the ONL in the survivor zone (highlighted by arrows). B' and D' are high magnification views of the rectangular areas in B and D, scale = 15 μm (**A**-**D**) or 6 μm (**B**', **D**').

In control retinas, PKCα staining revealed fine, bushy dendritic processes of rod bipolar cells only in the OPL (Figure 3A), while rod bipolar cells in LIRD retinas exhibited dramatic neuritogenesis occurring by pLX7 in the survivor zone (data not shown) and becoming more obvious by pLX30. Rod bipolar cells in the LIRD retinas extended their dendrites from the OPL into outer nuclear layer (ONL) (Figure 3B and 3B', highlighted by arrows) in the survivor zone. Further, calbindin immunostaining revealed calbindin-positive horizontal cells undergoing similar neuritogenesis. While neurites of horizontal cells were confined in the OPL in the control retina (Figure 3C), horizontal cells in the LIRD retinas extended their neurites from the OPL into the ONL in the survivor zone (Figure 3D and 3D', highlighted by arrows). These findings are consitent with the formation of novel neurites found in human RP, AMD and animal models of retinal degenerations [5,6,13,41-43].

### GluR2 and its trafficking proteins are expressed in the new neurites in the survivor zone

**Figure 4:**
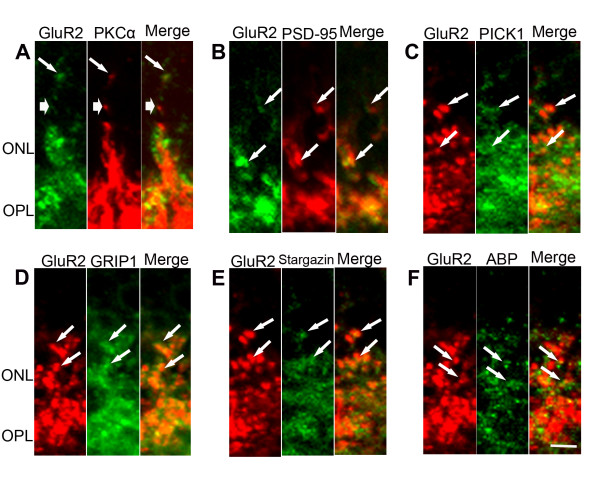
**GluR2 and its trafficking proteins were expressed in the new neurites in the survivor zone ONL**. **A**. GluR2 partially co-localized PKCα in the new neurites. Long tail arrows indicate colocalization of GluR2 and PKCα, while short tail arrows indicate non colocalization of GluR2 and PKCα in the new dendrites. **B**. GluR2 co-localized with PSD-95. **C**. GluR2 co-localized with PICK1. **D**. GluR2 co-localized with GRIP1. **E**. GluR2 co-localized with stargazin, and (**F**) GluR2 co-localized with ABP in the new neurites; scale = 3 μm.

We correlated the findings of neuritogenesis here with alterations in GluR2 trafficking. Examination of expression patterns of GluR2 and its trafficking proteins in new neurites demonstrated that GluR2, PSD-95, stargazin, PICK1, GRIP1 and ABP were present in the OPL as well as in the ONL during LIRD (Figure 4A-F). GluR2 partially co-localized with PKCα in the new neurites (Figure 4A), suggesting that GluR2 is expressed in the new neurites of rod bipolar cells during retinal degenerations. Different from PKCα, PSD-95 co-localized well with GluR2 in the new neurites in the ONL (Figure 4B). GluR2 also co-localized well with PICK1 (Figure 4C), GRIP1 (Figure 4D), stargazin (Figure 4E), and ABP (Figure 4F) in the new neurites in the ONL. These data are consistent with the data proposing PSD-95, stargazin, PICK1, GRIP1 and ABP as anchoring of GluR2 at synaptic locations [44].

### KIF3A interacts with GRIP1 and PSD-95 in mouse retina

**Figure 5:**
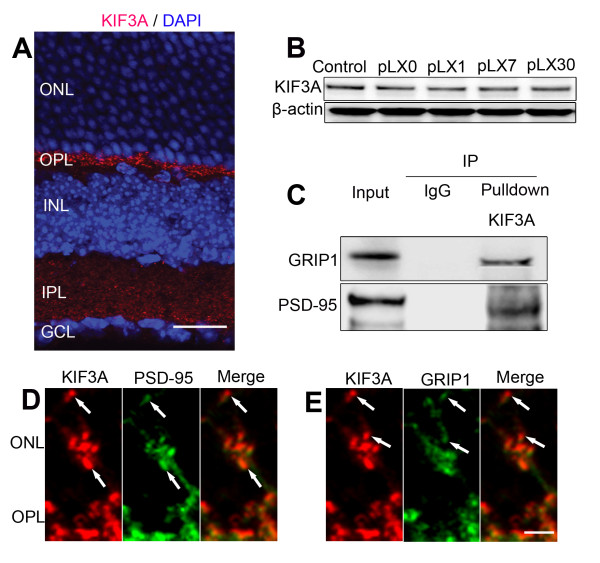
**KIF3A interacted with GRIP1 and PSD-95 in mouse retina**. **A**. KIF3A staining showed that KIF3A was expressed in the OPL and IPL (postnatal day 80). **B**. Western blotting analysis showed that LIRD had no obvious effect on the protein levels of KIF3A. **C**. Immunoprecipitation analysis showed direct interaction between KIF3A and PSD-95, GRIP1. **D**. **E**. Confocal imaging showed that KIF3A co-localized well with PSD-95 (**D**) and GRIP1 (**E**) in the new neurites in the ONL; scale = 15 μm (**A**) or 3 μm (**D**, **E**).

KIF3A is a reported motor protein expressed at the basal body of the connecting cilium axoneme and at the synaptic ribbons of cones and rods [45]. Our data showed that KIF3A was expressed in the OPL and IPL of control retina (Figure 5A), thereby raising the possibility that KIF3A interacts with postsynaptic density proteins which are also present in the OPL and IPL. Protein expression of KIF3A during LIRD was relatively stable, with a slight decrease on pLX7 (Figure 5B). Interactions between KIF3A and GRIP1 and PSD-95 were demonstrated by immunoprecipitation analysis (Figure 5C). During LIRD, KIF3A was also expressed in the new neurites, where KIF3A co-localized well with PSD-95 (Figure 5D) and GRIP1 (Figure 5E), suggesting that KIF3A may be involved in guiding GluR2 and its trafficking protein to the new dendrites.

### GluR2 and its trafficking proteins are lost in the light-damage zone

**Figure 6:**
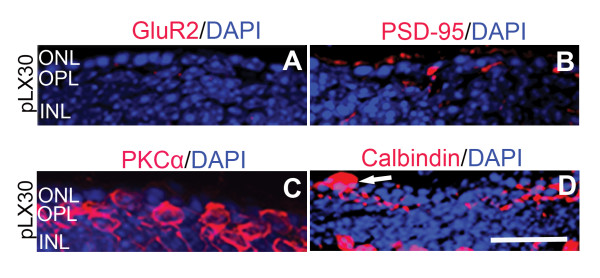
**GluR2 expression was lost in the light-damage zone**. Expression of GluR2 (**A**) and its trafficking proteins such as PSD-95 (**B**) were dramatically decreased in the OPL of the light-damage zone (pLX30). PKCα (**C**) and calbindin (**D**) staining showed that rod bipolar cells dendrites and horizontal cells neurites were truncated in the OPL the light-damage zone (pLX30); scale = 20 μm.

In contrast to the survivor zone, GluR2 expression disappeared by pLX30 in the light-damage zone, where only one layer of photoreceptors was left (Figure 6A). The expression of PSD-95 was also dramatically decreased in the light-damage zone (Figure 6B). Similar to GluR2, its trafficking proteins GRIP1, ABP, PICK1, and stargazin disappeared in the light-damage zone (data not shown). In parallel with the loss of GluR2 and its trafficking proteins, dendrites of rod bipolar cells and neurites of horizontal cells in the light-damage zone OPL were retracted and flattened (Figure 6C and 6D). We also observed a horizontal cell body located adjacent to the RPE at the distal border of the remnant ONL (Figure 6D, arrow).

### Protein levels of calcium-buffering proteins show no change during LIRD

**Figure 7:**
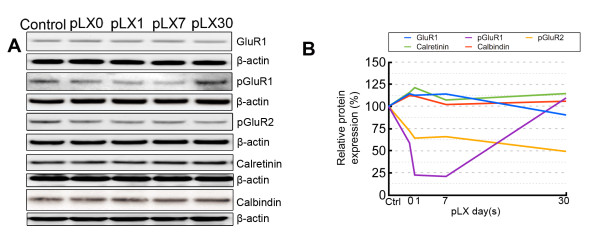
**Effect of LIRD on the protein expression of calcium-buffering proteins**. Western blotting (**A**) and analysis (**B**) showed that LIRD had no effect on the levels of GluR1, calretinin and calbindin, but decreased the levels of pGluR1 and pGluR2, n = 9.

The major subunits of AMPA receptors, GluR1 and GluR2, play have important roles in neuroplasticity [44]. Given that LIRD increased the levels of GluR2, we examined the effect of LIRD on GluR1. Phosphorylation of GluR1 is a major mechanism for controlling excitation. Since our light damage protocol spans the period of photoreceptor stress (increased extracellular glutamate) all the way to complete photoreceptor loss, we deemed it appropriate to screen events known in CNS to modulate excitation. Further, GluR2 binds to PICK1 after phosphorylation at serine 880 by PKCα, which induces dissociation of GluR2 from GRIP and the subsequent internalization of GluR2 by PICK1 for recycling or degradation [38]. Thus, we also measured the levels of phosphorylated GluR1 (Ser831) (pGluR1) and phosphorylated GluR2 (Ser880) (pGluR2). Our results revealed that light stress had no significant effect on the protein levels of GluR1 (Figure 7A and 7B). However, light stress rapidly decreased the levels of pGluR1, which showed an obvious decrease on pLX0 and reached a minimum on pLX7 but recovered by pLX30. Light stress also decreased the expression of pGluR2 by pLX0, extending its decrease by pLX30. Two calcium binding proteins, calretinin and calbindin, were also examined but showed no LIRD-induced change in protein expression (Figure 7A and 7B).

### Effect of AMPA receptors antagonist NBQX on neuritogenesis during LIRD

**Figure 8:**
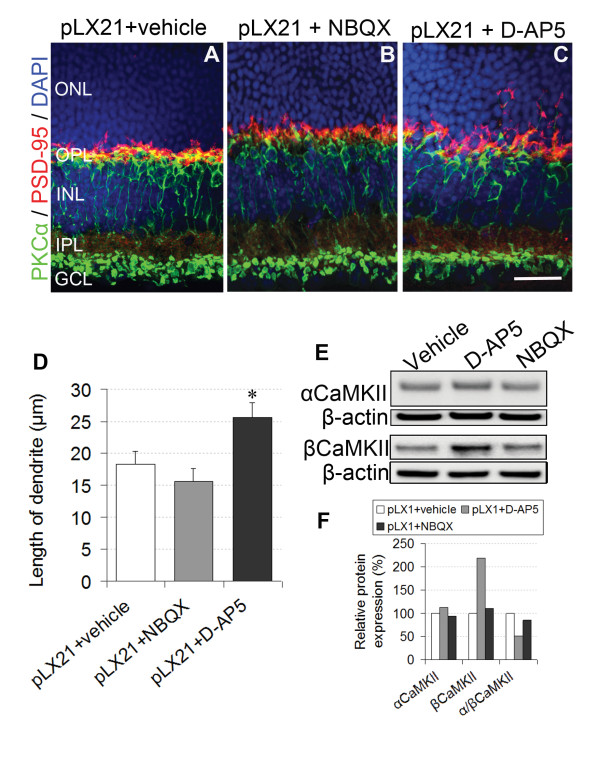
**AMPA receptors antagonists NBQX had no significant effect on neuritogenesis**. **A**-**C**. Effect of NBQX and D-AP5 on neuritogenesis. Compared with control (vehicle) (**A**), sub-retinal injection of NBQX (**B**) had no effect on neuritogenesis while D-AP5 (**C**) accelerated neuritogenesis. **D**. Summary data (mean ± s.e.m.) for the length of rod bipolar cell dendrites, *P < 0.05 vs control (vehicle), n = 5. E, F. Western blot (**E**) and analysis (**F**) showed the levels of αCaMKII and βCaMKII after sub-retinal injection of NBQX and D-AP5 (pLX1).

Ca^2+^/calmodulin-dependent protein kinase II (CaMKII) senses cytosolic Ca^2+ ^fluxes, which are largely mediated by glutamate-activated AMPA or NMDA receptors in CNS neurons [46]. CaMKII plays a central role in synaptic plasticity, both α- and β-CaMKII are neuron-specific and expressed in retinal neurons [47]. αCaMKII is activated by high Ca^2+ ^levels, while βCaMKII is more sensitive to lower Ca^2+ ^levels, and heteromeric CaMKII Ca^2+ ^responsivity and activity depends on the α/β subunit ratio [48]. Decreases in neuronal activity decrease the CaMKII α/β ratio, likely by upregulating transcription of βCaMKII [49], which positively correlates with the increase in low calcium permeability GluR2 expression in our study. Our previous study revealed that CaMKII plays an important role in neuritogenesis during retinal degeneration [8]. Therefore, we studied the effects of AMPA receptors antagonist NBQX on CaMKII signaling and neuritogenesis. Our results showed that NBQX slightly mitigated neuritogenesis. Consistently, NBQX had no effect on the ratio of α/βCaMKII compared with vehicle group during LIRD (Figure 8A, B, and 8D-F). However, NMDA receptors antagonist D-AP5 obviously increased the ratio of α/βCaMKII and significantly accelerated neuritogenesis (Figure 8A, C and 8D-F). Because NBQX is neuroprotective [50] but does not block downregulation of GluR2 [51], our results suggest that NBQX affords neuroprotection by a direct block of GluR2-lacking, Ca^2+^-permeable AMPA receptors [31]. Due to that GluR1 levels were not altered during LIRD, the above results totally suggest that AMPA receptor GluR2 in particular drives CaMKII signaling associated with neuritogenesis during LIRD.

## Discussion

We reveal a correlation of neurite formation and retraction with changes in expression of GluR2 and GluR2 trafficking machinery in the LIRD model. Alterations in GluR2 expression may provide a feedback mechanism for altering Ca^2+ ^permeability in deafferented retinal neurons, therefore be responsible for de novo neuritogenesis, and ultimately contribute to functional reprogramming of neural circuitry.

Normally, increasing the ratio of GluR2 subunits in iGluRs plays a protective role by decreasing Ca^2+ ^loads in neurons, which likely can not be blocked by AMPA receptors inhibitor NBQX [31]. Additionally, GluR2-mediated decreases in Ca^2+ ^permeability can prevent GluR1 phosphorylation-dependent increases in calcium conductance [52]. In LIRD, GluR1 expression is not changed while GluR1 phosphorylation decreases and levels of calcium-buffering proteins calretinin and calbindin remain stable. Similarly, data from diabetic subjects showed significant retinal increases of GluR2 but not GluR1 immunoreactivity in the OPL and IPL [53-55]. GluR2 upregulation in various retinal disease models suggests that this may be a protective response to photoreceptor-mediated stress and deafferentation.

Nevertheless, GluR2 levels (mRNA) were found unchanged in *rd *mouse retina [56]. Most inherited degenerations are slow compared to light damage and their time profiles are complex [1,2]. Even the fast *rd *model requires 3 months for all photoreceptors to die and there is no known coherent stress period (at least we don't know if anyone has published such an analysis). Light damage has a very precise onset of a few hours. The 1995 Duvoison paper [56] does not specify the time point at which the 35S GluR ISH autoradiographic measurements were made. As shown by Jones et al. [13,57], Marc et al. [1,2,4,12], RD (including light damage) have complex time profiles and pass through three distinct phases: phase 1 (photoreceptor stress and early death), phase 2 (active photoreceptor death), phase 3 (remodeling after all photoreceptors are lost). Our examination of that paper suggests that the *rd *animals were adult, in which case they were in late phase 3 remodeling and the outer retina was completely decimated, lacking bipolar cell and horizontal cell dendrites. The purpose of our current paper was to profile the kinetics of glutamate receptors before such a pathologic state. Indeed our results show that the major changes in GluR expression occur acutely during phase 1 of light damage and quickly normalize, suggesting that GluR2 modulation is acute.

GluR2-mediated changes in Ca^2+ ^permeability may be also responsible for the subsequent neurite outgrowth of retinal neurons. In embryonic chick retinal neurons, early activation of Ca^2+^-permeable AMPA receptors reduces neurite outgrowth [58], and influx of Ca^2+ ^through these channels could be responsible for the reduction in neurite outgrowth of cultured hippocampal pyramidal cells [59]. GluR2 co-localizing with its trafficking proteins PICK1, ABP, GRIP1 and stargazin at new neurites during LIRD suggests that this same mechanism may be represented in deafferented retina. The interaction of stargazin with AMPA receptors is essential for delivering functional receptors to the surface membrane, whereas binding with PSD-95, a GluR-clustering molecule, is required for targeting the AMPA receptors to synapses [60]. During GluR2 trafficking, the PICK1-ABP/GRIP interaction targets PICK1-PKCα complexes to ABP/GRIP-AMPA receptor complexes. GluR2 binds to PICK1 after phosphorylation at serine 880 by PKCα, which induces dissociation of GluR2 from GRIP and the subsequent internalization of GluR2 by PICK1 for recycling or degradation [38]. Therefore, rapid alterations in GluR2 and its trafficking during early LIRD may reveal mechanisms to limit Ca^2+^-mediated damage or simply reflect loss of dendritic iGluRs as dendrites are lost [4,13]. Normalization of GluR2 expression by pLX30 may reveal new GluR2 expression in novel dendrites that are in the process of forming new synaptic complexes.

This study demonstrates that expression of GluR2 and its trafficking proteins in the new dendrites may be guided through KIF3A, a member of the heteromeric family of kinesins which constitute a large family of microtubule motor proteins [45]. In neurons, kinesin motors conduct transport to axons and neurotransmitter receptors to dendrites and GRIP1 directly interacts with kinesin enabling transport of dendritic proteins such as GluR2 [61]. Our work reveals the expression of KIF3A in the OPL, where is dominated by ribbon synapses between presynaptic photoreceptors and postsynaptic horizontal cells and bipolar cells, both of which are involved in early reactive neuritogenesis in the degenerative retina. Interaction between KIF3A and GRIP1 and PSD-95 indicates that KIF3A has the potential to steer GRIP1 and PSD-95 to the new dendritic formation. Because GluR2 and its trafficking proteins are anchored with PSD-95, it is possible that GluR2 and its trafficking proteins are guided indirectly by PSD-95 and GRIP1 to the new processes.

In normal tissues, differential expression of GluRs by bipolar cells creates parallel ON and OFF channels in vision. ON bipolar cells express mGluR6 receptors while OFF bipolar cells express either AMPA or KA receptors. In retinal degenerative diseases, the observation of GluR2 in pathological newly-formed bipolar cell dendrites is a new finding and likely contributes to the functional reprogramming observed in bipolar cells of degenerative retina [4,13]. Altered differential expression of GluRs by bipolar cells explains the phenotypic revision and reprogramming of bipolar cells observed in RD [7,12,13]. Previously, we demonstrated phenotypic plasticity in human RP and in animal models of cone-sparing RP where loss of rods, but not cones, leads to phenotype revision, inducing a switch from ON to OFF phenotypes in rod bipolar cells [4]. Nevertheless, primate ON bipolar cells also express a cohort of AMPA receptor genes in normal retina [62] and GluR2 subunits have long been identified in rod bipolar cells [21,26,28,55,63]. The fact that normal rod bipolar cells express iGluR subunits argues that such switching may be within the normal capacity of bipolar cells [4].

## Conclusions

In summary, this work is the first to study GluR2 and its trafficking proteins during RD. Our findings suggest that rapid alteration of GluR2 trafficking/regulation is involved in pathological neuritogenesis and is a potential mechanism behind phenotypic reprogramming of retinal neurons. Modulation of GluR2 expression and its trafficking may slow down primary neurite remodeling or limit neuritogenesis. However, further research is needed to separate the GluR2-mediated pathways that contribute to neuroprotection and pathological neuritogenesis.

## Methods

### Animals

Age-matched female Balb/C albino mice (The Jackson Laboratory, Bar Harbor, ME) were maintained in dim light (20-40 lux) on a 12-12 cycle in normal phase (lights on 7:00-19:00) with ad libitum access to food and water. All experimental procedures were designed to minimize animal number and suffering, and were conducted with approval of the Institutional Animal Care Committees at the University of Utah in accordance with the ARVO Statement for the Use of Animals in Ophthalmic and Vision Research.

### LIRD and subretinal injections

LIRD and subretinal injections were performed as described previously [8]. In brief, mice were placed into the light-damage chamber, where the visible light intensity ranged from 2,500 to 3,000 lux, for 24 h by excluding one normal night cycle. Following post-light exposure (pLX), all animals were returned to the dim cyclic light environment and maintained for 0, 1, 7, and 30 days (pLX0, pLX1, pLX7, and pLX30). Under ketamine-xylazine anesthesia, 5 μM NBQX (Sigma, St. Louis, MO, USA) and 50 μM D-AP5 (Sigma) were injected into the sub-retinal space (central region) of albino mice in 0.5 μl volumes using a 33-gauge micro syringe (Hamilton) 30 min before light exposure. A successful sub-retinal injection caused swelling of the retina. Control retinas were injected with vehicle (DMSO) (Sigma).

### Sample processing

At the end of the experiment, eyes were rapidly harvested following decapitation under isoflurane anesthesia. For Western blotting analysis, retinas were dissected in Hank's balanced salt solution (HBSS, GIBCO, Carlsbad, CA, USA), then placed on ice in 1.5 ml tubes with RIPA buffer (RIPA lysis buffer kit, Santa Cruz, CA, USA) and homogenized using a sonicator (Fisher Scientific, Pittsburgh, PA, USA) three times for 12 sec each with 12 sec breaks between cycles. The samples were put on ice for 10 min, then centrifuged at 14,000 rpm at 4°C for 10 min. Supernatants were transferred to new tubes. The concentrations of protein in the samples were measured using the BCA assay (Pierce, Rockford, IL USA). For immunohistochemical analysis, whole eyes were removed and rinsed in HBSS, fixed in 4% paraformaldehyde (PFA) (Sigma) for 2 h at 4°C, and then washed with PBS (in g/L: NaCl 8, KCl 0.2, Na_2_HPO_4 _1.44, KH_2_PO_4 _0.24; pH 7.4) twice for 10 min each. Eyes were incubated in PBS with 20% sucrose for 4 h at 4°C. Serial 12 μm coronal sections were made with a cryostat microtome (Leica CM3050 S, Wetzlar, Germany) and collected on Superfrost/plus microscope slides (Fisher Scientific).

### Immunoprecipitation

Retinas were lysed in RIPA buffer (RIPA lysis buffer kit, Santa Cruz) for 1 h at 4°C with gentle agitation. Lysates were immunoprecipitated for 1 h at 4°C using KIF3A antibody (1:1000) and then protein G agarose beads (Roche, Indianapolis, IN, USA) [8]. Samples were analyzed by Western blotting.

### Western blotting analysis

Samples of entire group were pooled. Protein samples (~20 μg protein) were combined with NuPAGE LDS sample buffer (4×) (Invitrogen, Carlsbad, CA, USA) and NuPAGE sample reducing agent (10×) (Invitrogen), then boiled for 10 min at 95°C. Western blotting analysis was carried out using NuPAGE 4-12% Bis-Tris gels (Invitrogen) at 200 V for 40 min. Gels were electro-blotted onto PVDF membrane (Millipore, Bedford, MA, USA) for 1 h at 25 V using a wet electro-blotting system (XCell SureLock Mini-Cell, Invitrogen). Blots were blocked for 1 h in PBS with 0.1% Triton-x100, pH 7.4 (PBST) containing 5% non-fat dry milk (NFDM). Blots then were incubated overnight at 4°C in primary antibodies diluted in 5% NFDM-PBST solution (GluR2, 1:1000, Chemicon; pGluR2, 1:1000, Millipore; protein kinase C α (PKCα), 1:5000, Sigma; GluR1, 1:2000, Upstate; pGluR1, 1:1000, Millipore; PSD-95, 1:500, Chemicon; protein interacting with C kinase 1 (PICK1), 1:400, Santa Cruz; GRIP1, 1:1000, Millipore; stargazin, 1:500, Chemicon; AMPA receptor binding protein (ABP), 1:400, Santa Cruz; KIF3A, 1:1000, Covance; calretinin, 1:2000, Abcam; calbindin, 1:2000, Abcam; αCaMKII 1:1000, Upstate; βCaMKII: 1:1000, Invitrogen). Blots were washed three times for 10 min in PBST, incubated for 2 h in secondary antibodies (IgG-HRP, Santa Cruz; 1:5000 in 5% NFDM-PBST) followed by three more washes of 10 min in PBST. Immunostaining was revealed by the SuperSignal West Dura Extended Duration Substrate kit (Thermo Scientific, Waltham, MA, USA), and scanned using the Quantity One imaging system (Bio-Rad). Densitometry for each band was measured using ImageJ (U.S. National Institutes of Health, Bethesda, MD, USA). β-actin (1:5000, Sigma) was used as a loading control.

### Immunohistochemistry

Cryosections were washed 2 × 10 min in PBS, then blocked with blocking buffer for 30 min. Cryosections were incubated overnight at 4°C in primary antibodies diluted in blocking buffer (GluR2, 1:500, Chemicon or 1:200, Santa Cruz; PKCα, 1:2000; PSD-95, 1:500; PICK1, 1:200; GRIP1, 1:500; stargazin, 1:500; ABP, 1:100; KIF3A, 1:500; calbindin, 1:1000). These primary antibodies were from different species in every case and they did not cross-react on Western Blots. After washing 3 × 10 min in PBS, sections were incubated for 45 min at room temperature in secondary antibodies (cy3-, 488- or 647-conjugated IgG, Invitrogen) diluted 1:1000 in blocking buffer. For double or triple staining, sections were sequentially incubated with primary antibodies and secondary antibodies as above. After incubation with antibodies, sections were washed 3 × 10 min in PBS, then treated with 10 μM DAPI (Invitrogen) for 5 min at room temperature. Sections from all groups were processed simultaneously to reduce staining artifacts or intensity differences. Negative controls were performed by omission of the primary antibodies.

### Confocal imaging

Fluorescent images were acquired with an Olympus FV1000 laser-scanning confocal microscope (Olympus, Tokyo, Japan). Settings were chosen so that pixel intensities for the brightest samples were just below saturation, except when the processes of retinal neurons had to be clearly determined, in which case signals from certain areas (soma of the retinal neurons) were saturated in order to obtain a clear perimeter of the neurites. Optical slice units were 0.5 μm. Neuritogenesis was analyzed as described [8]. In brief, neurite length was measured from the point of emergence at the cell body to the tip of each segment. One section was selected from each animal, and 20 longest dendrites from 20 rod bipolar cells in a specific region (ventral mid-peripheral region, 120 μm in length) were measured and calculated as the mean. Quantification of the morphological parameters was carried out using ImageJ by investigators blinded to experimental conditions.

### Data analysis

Data of neuritogenesis were expressed as mean ± SD and analyzed with SPSS 12.0 (SPSS Inc.). Statistical comparisons were made using Bonferroni tests and analysis of variance (ANOVA), P < 0.05 was defined as the level of significance. Protein levels represented pooled data of entire groups and were expressed as means only. To study the rationality of pooling samples in our experiment, we actually set up 3 batches of animals. There were 3 mice per group in each batch (i.e. 9 mice in total for each group). We pooled the 3 mice's retinas from each group in each batch and ran the western blotting for protein analysis. It was found that the biological variations for the 9 mice were low and GluR2 increase, for example, was significant during early LIRD (Additional file 1 Figure S1).

## Abbreviations

LIRD: light-induced retinal degeneration; AMD: age-related macular degeneration; RP: retinitis pigmentosa; pLX: post-light exposure; GluRs: glutamate receptors; GluR2: glutamate receptor 2; pGluR2: phosphorylation of glutamate receptor 2; CaMKII: Ca^2+^/calmodulin-dependent protein kinase II; PKCα: protein kinase C α; GRIP1: glutamate receptor-interacting protein 1; PSD-95: postsynaptic density protein 95; KIF3A: kinesin family member 3A; ABP: AMPA receptor binding protein; PICK1: protein interacting with C kinase 1; RPE: retinal pigment epithelium; OPL: outer plexiform layer; ONL: outer nuclear layer; INL: inner nuclear layer; AMPA: α-amino-3-hydroxy-5-methyl-4-isoxazolepropionic acid; NMDA: N-methyl-D-aspartate.

## Competing interests

The authors declare that they have no competing interests.

## Authors' contributions

YHL conceived and designed experiments, either performed or participated in all experiments, analyzed data, and wrote the manuscript; REM conceived experiments, analyzed data, and revise the manuscript; BWJ and AHL performed LIRD, analyzed data and revised the manuscript; FRV, JSL and WDF revised the manuscript. All authors have read and approved the final manuscript.

## Supplementary Material

Additional file 1 **Figure S1**. Rationality of pooling samples in our experiment. Click here for file
