# Supplementary material for: Rapid glutamate receptor 2 trafficking during retinal degeneration
Source: Mol Neurodegener. 2012 Feb 10;7:7. doi: 10.1186/1750-1326-7-7 (PMC3296582; doi:10.1186/1750-1326-7-7)
Supplement: Additional file 1 — Figure S1. Rationality of pooling samples in our experiment. [file 1750-1326-7-7-S1.PDF]

Additional file 1

Rapid glutamate receptor 2 trafficking during retinal degeneration

Yanhua Lin, Bryan W. Jones, Aihua Liu, Félix R. Vazquéz-Chona,
J. Scott Lauritzen, W. Drew Ferrell, Robert E. Marc

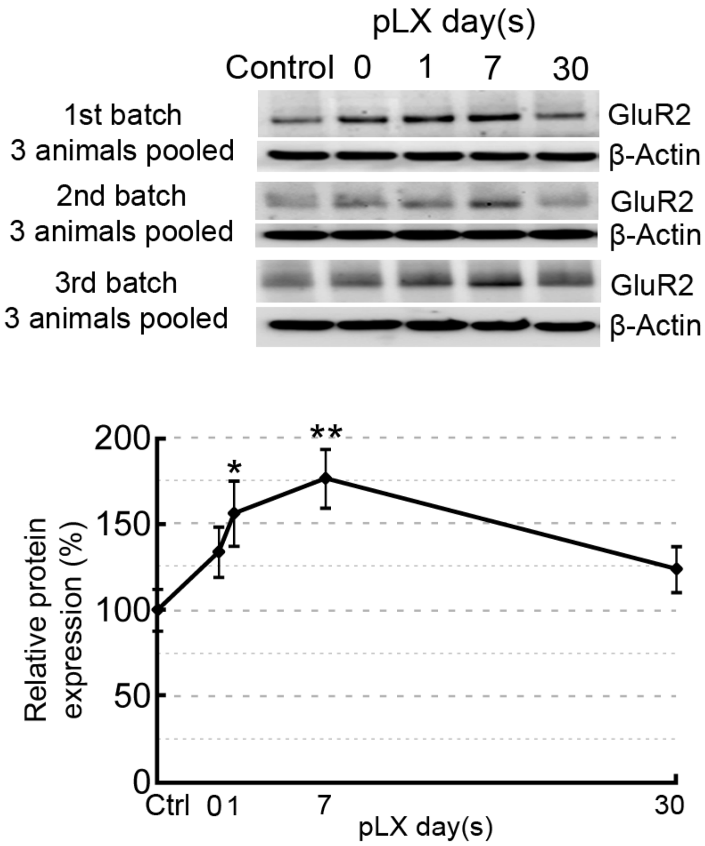

Additional file 1 Figure S1.

Rationality of pooling samples in our experiment. 3 batches of animals were set up. There were 3 mice per group in each batch (i.e. 9 mice in total for each group). We pooled the 3 mice's retinas from each group in each batch and ran the western blotting for protein analysis. It was found that the biological variations for the 9 mice were low and GluR2 increase, for example, was significant (Bonferroni tests) during early LIRD, *P<0.05, **P<0.01 *vs* control.
